# Editorial: Redox-Active Molecules as Antimicrobials: Mechanisms and Resistance

**DOI:** 10.3389/fmicb.2021.758750

**Published:** 2021-09-10

**Authors:** Jong H. Kim, Luisa W. Cheng, Kirkwood M. Land, Martin C. H. Gruhlke

**Affiliations:** ^1^Western Regional Research Center, Agricultural Research Service, Foodborne Toxin Detection and Prevention Research Unit, United States Department of Agriculture, Albany, CA, United States; ^2^Department of Biological Sciences, University of the Pacific, Stockton, CA, United States; ^3^Department of Plant Physiology, Rheinisch-Westfälische Technische Hochschule Aachen University, Aachen, Germany; ^4^GENAWIF e.V. – Society for Natural Compound and Active Substance Research, Aachen, Germany

**Keywords:** antimicrobials, drug resistance, mode of action, redox molecules, sulfur compounds

Current drugs for treating microbial infections have limited efficiency, especially for eliminating drug resistant pathogens. Although high-throughput screenings have been investigated for identifying novel antimicrobial drug candidates, stagnation in development of new, safe antimicrobial agents is a persistent public health concern (Tillotson and Tillotson, 2015). Recent investigations have determined that the antimicrobial mechanisms of certain drugs involve oxidative stress/damage in pathogens, and therefore, those drugs are further defined as oxidative stress drugs. For example, ciprofloxacin is a fluoroquinolone antibiotic inhibiting the function of DNA topoisomerases. Of note, when bacteria are treated with ciprofloxacin, the level of reactive oxygen species (ROS) increases in the pathogens. However, application of antioxidant molecules or transfection of superoxide dismutase gene into bacteria reversed fluoroquinolone toxicity (Goswami et al., 2006). Various sulfur compounds as natural substances belong to the class of reactive sulfur species and lead to a modification of cysteine protein residues by oxidative means, which leads to antimicrobial capability in pathogenic bacteria (for example, in the case of the natural substance allicin; Loi et al., 2019). The anti-parasitic drug albendazole (ABZ) is commonly used for treating the parasitic protozoan *Giardia duodenalis*. Besides binding to β-tubulin, ABZ also induces oxidative stress and DNA damage in *G. duodenalis* (Martínez-Espinosa et al., 2015). Amphotericin B (AMB) is a polyene antifungal drug that binds to ergosterol in cell membrane. While AMB binding results in membrane depolarization, alteration in permeability, and cellular leakage/rupture, induction of cellular oxidative damage is another antifungal action of AMB (Jukic et al., 2017).

ROS-generating antimicrobials is an important subject in antimicrobial resistance study, where modern sensors are employed to accurately analyze the levels of ROS generated by redox-active antimicrobials against different pathogens. For instance, to target the bacterial pathogen *Pseudomonas aeruginosa* via exogenous ROS generation system, a glycomimetic-decorated fluorescent nanobiocide was developed which mediates ROS release from the functionalized CuInS/ZnS quantum dots (Li et al., 2021). This system simultaneously fulfilled a fluorescent monitoring and sterilization, where the fluorescence intensity was determined at 525 nm by the microplate reader. The natural naphthoquinone lapachol induced oxidative stress (ROS formation) against *Staphylococcus aureus*, a human pathogen rapidly acquiring multidrug resistance. The level of oxidation by lapachol was determined using *S. aureus* expressing the biosensor plasmids (Linzner et al., 2020), where the biosensor fluorescence emission was measured at 510 nm; a similar biosensor was further applied against a mycoredoxin-null mutant of *Rhodococcus equi* (mammalian pathogen) to screen ROS-generating antibiotics exerting drug synergism (Mourenza et al., 2020). Meanwhile, the use of photosensitizers was investigated in photodynamic therapy against the yeast pathogen *Candida albicans*, where the production of ROS was determined using an electron paramagnetic resonance spectrometer (Kanpittaya et al., 2021). While all photosensitizers tested exhibited no toxicity on the fibroblast cells, selected photosensitizers inhibited *C. albicans* like the polyene drug nystatin. Further studies utilizing sensors to investigate ROS-generating antimicrobials include: (a) real-time single-cell fluorescence on *Escherichia coli* stained with a fluorogenic probe measuring lipid peroxyl radicals after ciprofloxacin treatment (Martínez et al., 2020), and (b) aggregation-induced emission (AIE) luminogens possessing high ROS generating capacity as a probe, which simultaneously detecting and performing photodynamic ablation of macrophage-engulfed Gram-positive bacteria (Lee et al., 2020).

In this Research Topic, six works (four original research articles, one review and one hypothesis and theory) were published on the use of redox-active molecules as antimicrobials, providing oxidative mechanisms and resistance management tools.

Thioredoxin reductase (TrxR) maintains systemic redox homeostasis in Gram-positive bacteria, limited number of Gram-negative bacteria and several parasites, hence TrxR can serve as a potential therapeutic target. In their Hypothesis and Theory paper, Felix et al. described that TrxR is a potential target for auranofin, ebselen, shikonin, and allicin, where auranofin is the most potent drug. Auranofin has been used as an anti-rheumatoid/arthritis drug but is recently repurposed to treat microbial infection. Auranofin interacts with TrxR in Gram-positive bacteria, where the CXXC motif could be the target. However, most Gram-negative bacteria can use another antioxidant system, glutathione-glutaredoxin (GSH), to scavenge reactive oxygen/nitrogen species and maintain redox homeostasis. GSH-independent pathogens, notably the clinically important *Mycobacterium tuberculosis* and Gram-negative *Helicobacter pylori*, remain susceptible to TrxR inhibitory drugs.

In a related study, Gong et al. investigated the effect of increased temperature on metronidazole resistance in *H. pylori*, where RNA-sequencing based transcriptomic profiling identified differentially expressed genes when the pathogen was grown at 41°C. Resistance to metronidazole was reduced at the higher temperature. Redox pathways seemed the potential drivers of metronidazole resistance at higher temperature; the expression of superoxide dismutase (*Sod*) gene was down-regulated at 41°C, and accompanied by the enhancement of a negative regulator of *Sod*. However, the transcriptional changes in TrxR system were not determined in the study.

The antibacterial mechanism of lysozyme-coated silver nanoparticles (AgNPs) was investigated in a multi-drug resistant *Klebsiella pneumonia* (Pareek et al.). AgNPs provide a greater surface area, leading to an enhanced controlled release of silver ion (Ag^+^) to the target. Biochemical and transcriptional analyses (RNA sequencing) revealed AgNPs induced a *triclosan-like* bactericidal effect, thus inhibiting the expression of the type II fatty acid biosynthesis genes. Of note, the released Ag ion triggered oxidative stress in *K. pneumonia*, where Δ*soxS* mutant exhibited increased susceptibility to AgNPs compared to the wild type.

The protozoan parasite *Trypanosoma cruzi* is a causative agent of Chagas disease. Although benznidazole is currently the main therapeutic agent, this drug causes severe side effects while possessing low efficacy against the chronic phase of the disease. Macedo et al. examined the trypanocidal efficacy of novel phenyl-*tert*-butyl-nitrone (PBN) derivate LQB303 against *T. cruzi*, where this compound exhibited a potent trypanocidal activity against intra-/extracellular amastigotes *in vitro*. LQB303 caused mitochondrial dysfunction, which resulted in impaired oxygen consumption and the spare respiratory capacity of amastigotes. Of note, one of the PBN derivatives *alpha*-phenyl-*N*-*tert*-butylnitrone (PBN) nitrone possesses free radical spin trap capacity, thus exhibiting low toxicity to the host.

Certain azole fungicides applied to crop fields have the same mode of antifungal action as clinical azole drugs. Hence, long-term application of azole fungicides to agricultural fields provides a selection pressure for the emergence of pan-azole-resistant fungal pathogens. Meanwhile, thymol is a redox-active secondary metabolite that can induce oxidative stress in pathogens or impair fungal antioxidant systems. Shcherbakova et al. recently investigated the chemosensitizing capability of thymol to the azole fungicides difenoconazole and tebuconazole. In both seed and foliar treatments, co-application of thymol with azole fungicides synergistically inhibited the growth of the phytopathogens *Bipolaris sorokiniana, Parastagonospora nodorum* and *Fusarium culmorum*, while thymol did not enhance the production of mycotoxins (deoxynivalenol, zearalenone). A chemosensitizer, such as thymol, causes the target pathogen to become more susceptible to the co-applied drug by negatively modulating the pathogen's defense system.

Finally, Li et al. reviewed the involvement of ROS in antibiotic- and host-mediated pathogen killing. For example, quinolones-induced killing of bacterial pathogens is accompanied by the generation of endogenous hydroxyl radicals; primary damage caused by antibiotics stimulates a pathway that leads to ROS accumulation as a secondary damage. Exogenous ROS are generated from NADPH oxidase *via* phagocytes in the host cells, and could directly kill pathogens. It remains unknown precisely how phagocytic ROS inhibits pathogen growth. Li et al. described exogenous ROS-induced killing depends on a variety of innate mechanisms.

In summary, the antioxidant system of microbes could be an effective target for pathogen control. Redox-active molecules (natural or synthetic) can function as potent redox-cyclers in microbes, which contributes to the inhibition of pathogen growth by disrupting cellular redox homeostasis or the function of redox-sensitive cellular components. Identification of new, safe redox-active molecules and elucidation of their oxidative mechanisms will further the control of microbial pathogens, especially those resistant to current therapeutic agents.

## Author Contributions

JK, LC, KL, and MG were joint co-editors of this Research Topic. All authors contributed equally to the article and approved the submitted version.

## Funding

JK and LC were supported by USDA-ARS CRIS Project 5325-42000-054-00D.

## Conflict of Interest

The authors declare that the research was conducted in the absence of any commercial or financial relationships that could be construed as a potential conflict of interest.

## Publisher's Note

All claims expressed in this article are solely those of the authors and do not necessarily represent those of their affiliated organizations, or those of the publisher, the editors and the reviewers. Any product that may be evaluated in this article, or claim that may be made by its manufacturer, is not guaranteed or endorsed by the publisher.
